# *Plasmodium falciparum* with *pfhrp2* and *pfhrp3* gene deletions in asymptomatic malaria infections in the Lake Victoria region, Kenya

**DOI:** 10.1186/s41182-024-00664-7

**Published:** 2024-12-18

**Authors:** Takatsugu Okai, Chim W. Chan, Achyut KC, Protus Omondi, Kelvin Musyoka, James Kongere, Wataru Kagaya, Gordon Okomo, Bernard N. Kanoi, Yasutoshi Kido, Jesse Gitaka, Akira Kaneko

**Affiliations:** 1 Department of Virology, Graduate School of Medicine, Osaka Metropolitan University, Osaka, Japan; 2 Department of Parasitology, Graduate School of Medicine, Osaka Metropolitan University, Osaka, Japan; 3 Department of Parasitology and Entomology, Muhimbili University of Health and Allied Sciences, Dar es Salaam, Tanzania; 4 Ubuntu Health, Atlanta, GA USA; 5 Department of Eco-Epidemiology, Institute of Tropical Medicine (NEKKEN), Nagasaki University, Nagasaki, Japan; 6 Homa Bay County Ministry of Health, Homa Bay, Kenya; 7 Centre for Malaria Elimination, Mount Kenya University, Thika, Kenya; 8 Island Malaria Group, Department of Microbiology, Tumor and Cell Biology (MTC), Karolinska Institutet, Stockholm, Sweden; 9 Osaka International Research Center for Infectious Diseases, Osaka Metropolitan University, Osaka, Japan

**Keywords:** Malaria, *Plasmodium falciparum*, Rapid diagnostic tests (RDTs), *pfhrp2*, *pfhrp3*, Gene deletions, Kenya

## Abstract

Malaria rapid diagnostic tests (RDTs) targeting the *Plasmodium falciparum* histidine-rich protein 2 (*Pf*HRP2) are widely used to diagnose *P. falciparum* infection. However, reports of *P. falciparum* strains lacking *Pf*HRP2 and the structurally similar *Pf*HRP3 have raised concerns about the utility and reliability of *Pf*HRP2-based RDTs. This study investigated the presence of *P. falciparum* with *pfhrp2* and/or *pfhrp3* gene deletions among infected residents in the Lake Victoria region, Kenya. Four cross-sectional malaria, surveys were conducted in four sites (Suba South, Mfangano, Kibuogi, and Ngodhe) from September 2018 to January 2020. *P. falciparum* infections were detected using a *Pf*HRP2-based RDT, microscopy, and PCR on 9120 finger-prick blood samples. Samples negative by RDT but positive by PCR were selected for PCR amplification of *pfmsp1* and *pfmsp2* to confirm the quality and quantity of *P. falciparum* DNA. Samples positive for both *pfmsp1* and *pfmsp2* were included for detection of deletions of exons 1 and 2 in *pfhrp2* and *pfhrp3* PCR. The multiplicity of infection (MOI) was determined as the higher allele count between *pfmsp1* and *pfmsp2*. Logistic regression analysis was performed to analyze the association between *pfhrp2* and/or *pfhrp3* deletions and demographic and infection variables. Of the 445 RDT-negative and PCR-positive samples, 125 (28.1%) were analyzed for *pfhrp2* and *pfhrp3* deletions. Single *pfhrp2* deletion, single *pfhrp3* deletion, and *pfhrp2/3* double deletions were detected in 13 (10.4%), 19 (15.2%), and 36 (28.8%) samples, respectively. Single *pfhrp2* deletion was found in all sites while single *pfhrp3* deletion was found in all sites except Kibuogi. The majority of samples with *pfhrp2* and/or *pfhrp3* deletions were submicroscopic (73.5%), asymptomatic (80.9%), and monoclonal (80.9%). Polyclonal infection was significantly (*p* = 0.022) associated with a lower odds of *pfhrp2/3* double deletion, suggesting detection of intact *pfhrp2/3* in mixed infections. We report the presence of *P. falciparum* with *pfhrp2/pfhrp3* double deletions among asymptomatic and submicroscopic infections in Kenya. Our findings highlight the need for active monitoring of *pfhrp2* and *pfhrp3* deletions at the community level to improve malaria detection and control in the region.

**Supplementary Information:**

The online version contains supplementary material available at 10.1186/s41182-024-00664-7.

## Background

Malaria is a major vector-borne parasitic disease. In 2022, approximately 233 million malaria cases and 580,000 malaria deaths were reported in sub-Saharan Africa (SSA), accounting for 94 and 95% of global malaria cases and deaths, respectively [1]. Among the species that infect humans, *Plasmodium falciparum* causes the most serious health complications and is responsible for most deaths each year [1]. Malaria rapid diagnostic tests (RDTs) and light microscopy are used to diagnose malaria infection. In the last two decades, RDTs use has increased, since it requires no equipment and little training and can provide results in about 15 min [2]. The improved sensitivity and specificity of RDTs have enhanced the early diagnosis and treatment of malaria [3–5].

Malaria RDTs are lateral flow immunochromatographic antigen detection devices. Dye-labeled antibodies first bind to the parasite antigen in blood, and the resulting complex is captured on the nitrocellulose strip by a band of bound antibodies, forming a visible line in the resulting window that signifies a positive diagnosis [6]. Most RDTs detect two antigens: *Plasmodium* lactate dehydrogenase (*p*LDH), which is present in all *Plasmodium spp.* that infect humans, and *P. falciparum* histidine-rich protein 2 (*Pf*HRP2), which is present in *P. falciparum* only [7, 8]. *Pf*HRP2-based RDTs primarily detect the product of the *pfhrp2* gene on chromosome 8 but can also cross-react with the product of the *pfhrp3* gene on chromosome 13, due to extensive sequence homology between the two genes [9, 10].

In early 2000s, parasites with partial or total *pfhrp2* and/or *pfhrp3* deletions that escaped detection by *Pf*HRP2-based RDTs were first reported in Peru [11]. A subsequent analysis revealed that a large proportion of *P. falciparum* in the Peruvian Amazon harbored *pfhrp2/pfhrp3* double deletions, calling into question the continued utility of *Pf*HRP2-based RDTs in the country [12]. Currently, several malaria-endemic countries have reported parasites with *pfhrp2/pfhrp3* double deletions [13, 15, 16, 16–19]. These parasites are thought to spread preferentially, especially in areas where *Pf*HRP2-based RDTs are the only available diagnostic [20].

In SSA, *P. falciparum* causes over 95% of malaria cases. This has led to a preference for *Pf*HRP2-based RDTs, which are reported to be more sensitive and heat-stable than RDTs detecting other malaria antigens [7, 21]. However, *P. falciparum* with *pfhrp2* and/or *pfhrp3* deletions has been reported in a number of countries, including Eritrea [13, 22] and Kenya [9, 23, 24]. Most of the reports are based on samples obtained from symptomatic patients seeking care at health facilities. Yet, in highly endemic areas, most *P. falciparum* infections are asymptomatic [25], which raises the possibility that prevalence of *P. falciparum* with *pfhrp2/pfhrp3* double deletions could be underestimated, as asymptomatic individuals are often undetected and untreated and serve as parasite reservoirs for transmission. To address this knowledge gap, we investigated the presence of *pfhrp2* and/or *pfhrp3* deletions in *P. falciparum* among residents in Homa Bay County, Kenya, a region with high malaria endemicity bordering Lake Victoria [26].

## Methods

### Ethics statement

Ethical approval was provided by the Kenyatta National Hospital/University of Nairobi Ethical Research Committee in Kenya (No. P7/1/2012), the Mount Kenya University Independent Ethical Research Committee (MKU–IERC; approval No. 1574, 2848 and 2565), and the Ethics Committee of Osaka Metropolitan University (approval No. 3206).

Consent forms detailing the purpose, procedure, benefits and potential risks were distributed to students at least 1 day before the survey. The students were asked to request their parents or guardians to read and sign their consent forms. Only students who provided signed consent forms from their parents or guardians were included. Verbal assent was obtained from each student at enrollment. For adult participants, written informed consent was provided at enrollment.

### Characteristics of the study area

**Fig. 1:**
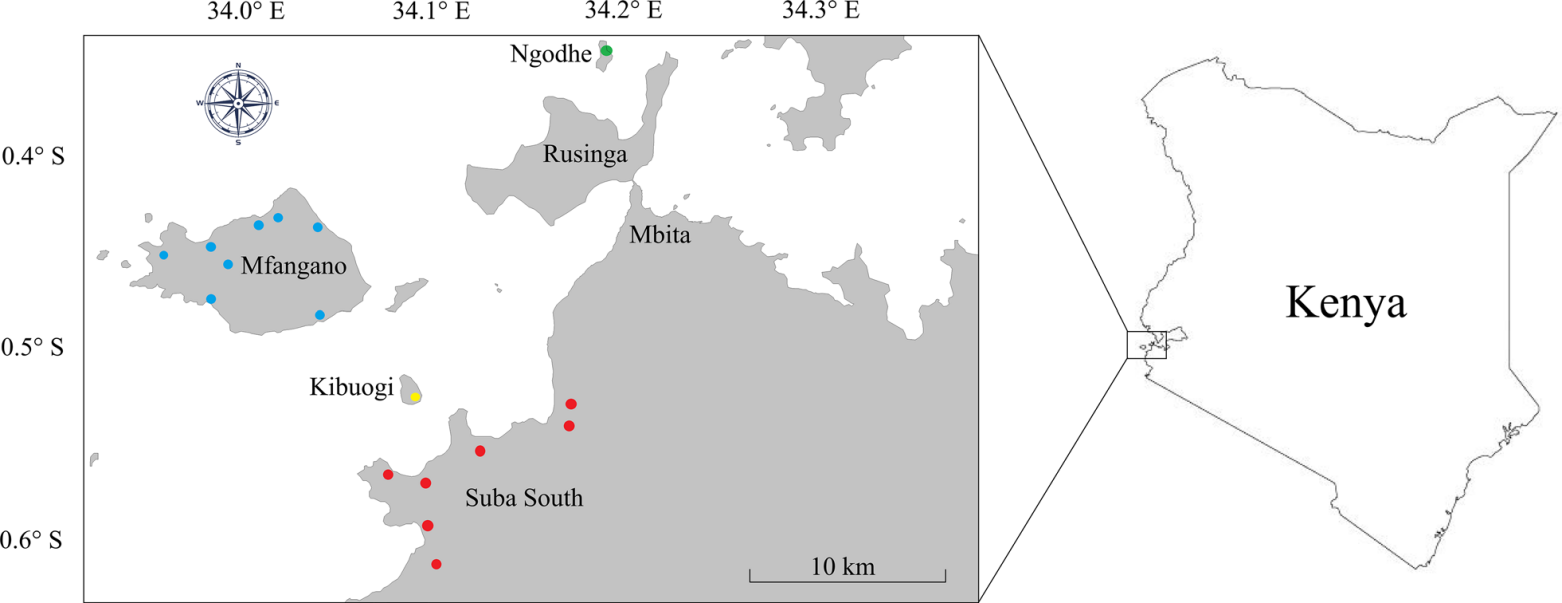
Study sites in Homa Bay County, Kenya 2018-2020. Inset shows the location of the study area. Approximate locations of schools are represented by colored points

The study was conducted in Suba South, Mfangano, Ngodhe, and Kibuogi in Homa Bay County in western Kenya bordering Lake Victoria (Fig. 1). Mfangano (65 km^2^) is a large, rural island with a population of 24,123 [27]. The island is connected to Mbita Town by scheduled ferry services and private motorboats. The island has ten public health facilities: five dispensaries, four health centers, and one hospital. Kibuogi (1.5 km^2^) and Ngodhe (0.8 km^2^) are small islands, each with a population of about 500 [28]. Kibuogi is connected to Mfangano and lakeshore communities in Suba South by private boats but has no public health facilities. Ngodhe is connected to Rusinga Island and Mbita Town by private boats and is served by one dispensary. Suba South has a population of 122,383 and consists of smaller villages such as Ungoye and Roo [27]. It is connected to Mbita Town by an unpaved road (Fig. 1).

All study sites are located in the Lake Endemic Zone, which has the highest malaria prevalence in Kenya [8], although significant local variations exist [29]. The zone generally experiences a long rainy season from March to June and a short rainy season from November to December, with some annual variations. Annual rainfall ranges from 250 to 1000 mm. Malaria incidence peaks approximately 1–2 months after the rainy season [26]. The main malaria vectors in the region are *Anopheles gambiae s.s.*, *An. arabiensis*, and *An. funestus* [30]. Vector control measures in Kenya include the mass distribution of long-lasting insecticidal nets (LLINs) every 3 years, which began in 2004. Since 2018, indoor residual spraying (IRS) with the organophosphate insecticide Actellic 300CS has been implemented in Homa Bay County annually, further reducing the malaria prevalence [31]. In addition, the RTS,S/AS01 malaria vaccine has been piloted in parts of the county since 2019 [32]. Currently, malaria diagnosis in most health care facilities within the study area relies on *Pf*HRP2-based RDTs, and artemisinin-based combination therapies (ACTs) remain efficacious in Western Kenya [33]. Despite these measures, persistent malaria transmission is maintained by asymptomatically infected individuals with submicroscopic parasitemia [31].

### Field and laboratory methods

We conducted four cross-sectional malariometric school surveys in the study sites in September 2018, January 2019, September 2019, and January 2020. Schools serving the main population centers in the study area were listed, after which approval from the school administrations to conduct our surveys was sought. In each population center, one to two schools that had provided approval were selected based on ease of access and availability that accommodated our survey schedule.

The primary purpose of our surveys was to determine the prevalence of *Plasmodium* infections in the study area. Our previous study [25] indicated local heterogeneity in *Plasmodium* prevalence, resulting in estimates of required sample sizes ranging from 145 to 344. From each selected school we obtained a list of enrolled students and selected a minimum of 150 children randomly. Since the populations of Kibuogi Island and Ngodhe Island were relatively small (approximately 500 each), the entire populations including adults were recruited. In January 2019 Ngodhe and Kibuogi were not included due to an ongoing intervention study, thus surveys were conducted on Mfangano and in Suba South only.

Demographic information including sex, age, and village of residence and self-reported LLIN use on the night before the survey were recorded for all study participants. Axillary body temperature was measured using digital thermometers, and fever was defined as axillary temperature ≥ 37.5 °C.

Finger-pricked blood sample was obtained for detection of *P. falciparum* infections by RDT, microscopy, and PCR in all years and locations. *P. falciparum* infections were diagnosed on-site using the *Pf*HRP2-based Paracheck *Pf* RDT (Orchid Biomedical Systems, Goa, India) according to the manufacturer’s instructions. Participants with positive RDT were given the standard course of artemether–lumefantrine treatment (and antipyretic treatments if the infection was accompanied with fever) with dosage instructions per recommendation by the Ministry of Health in Kenya.

Thin and thick blood films were prepared on site and transported to our field laboratory in Mbita Town. Thin films were fixed with methanol and both thick and thin films were stained with 3% Giemsa solution for 30 min. Stained blood films were examined independently by two experienced microscopists for *Plasmodium* infections and species identification [34]. A sample was declared negative after examination of at least 100 oil immersion microscopic fields at 1000X magnification [34]. Discrepant results were resolved by a third experienced microscopist who was blinded to results of the first two examinations. A finger-pricked blood sample (70 μl) was drawn using a 75-mm heparinized microhematocrit capillary tube (Thermo Fisher Scientific, MA, USA), spotted onto Whatman ET31 Chr filter paper (Whatman International, Maidstone, UK), and allowed to dry at ambient temperature. The dried blood spot (DBS) was placed in a small zipper plastic bag and stored at − 20 °C until DNA extraction.

### DNA extraction and PCR diagnosis

**Fig. 2:**
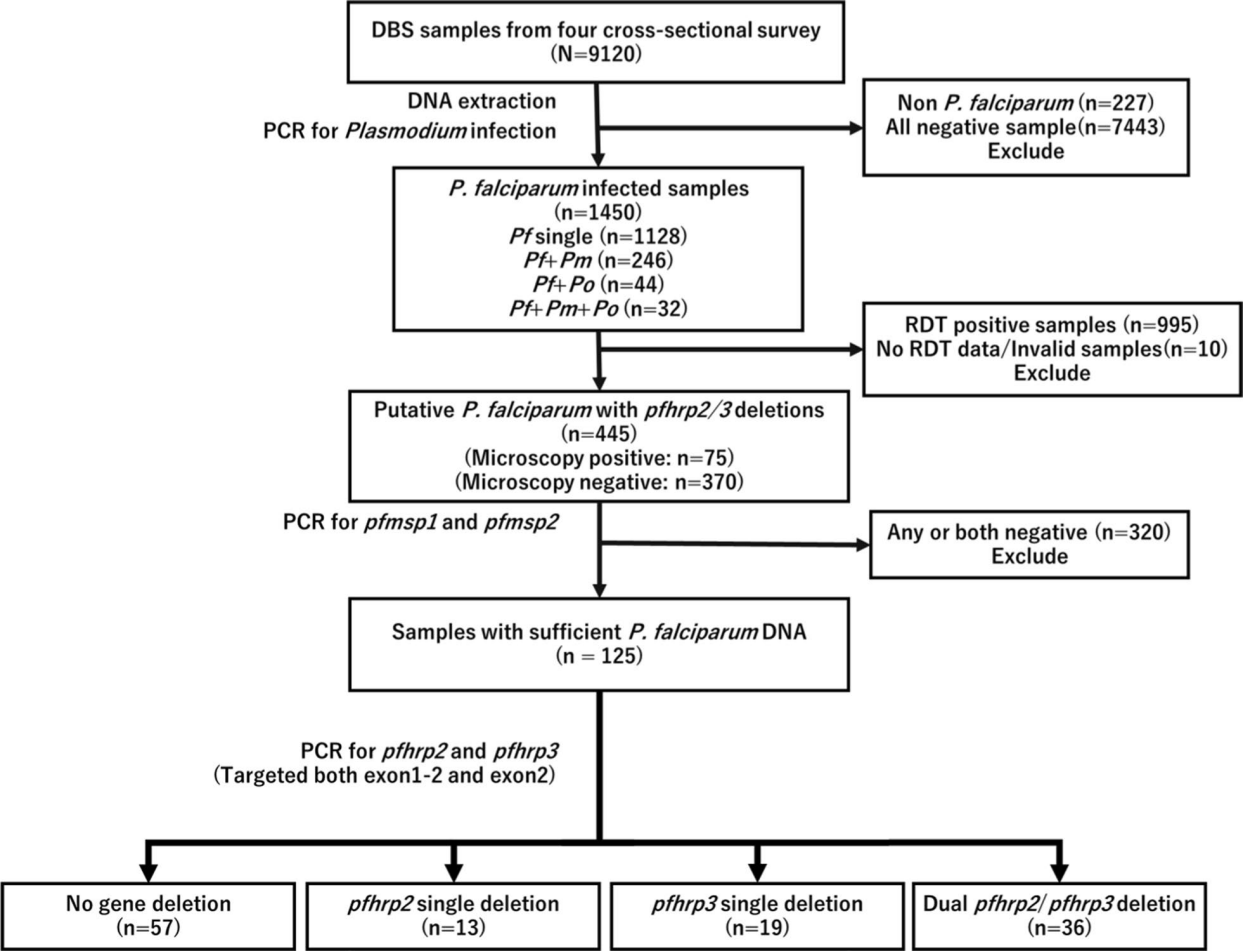
Flowchart of dry blood spots (DBS) selection for detection of *pfhrp2* and *pfhrp3* deletions

The procedure for sample selection is shown in Fig. 2. DNA extraction and PCR diagnosis were performed at Osaka Metropolitan University in Osaka, Japan. The QIAamp Blood Mini Kit (QIAGEN, Germantown, USA) was used to extract DNA from a quartered DBS (equivalent to 17.5 µl of blood) according to the manufacturer’s instructions. DNA was eluted in 150 µl of elution buffer (10 mM Tris–Cl and 0.5 mM EDTA; pH 9.0) and stored at − 20 °C. *Plasmodium* infections were detected by a nested PCR targeting the multi-copy mitochondrial cytochrome c oxidase subunit III (*cox3*) gene, using 3 µl of extracted DNA (equivalent to 0.35 µl of blood) as template [35].

### *P. falciparum* DNA quality control

To ensure the presence of *P. falciparum* nuclear DNA, all samples positive for *P. falciparum* by the *cox3* PCR but negative by RDT underwent DNA quality control by nested PCR amplification of the single-copy *P. falciparum* merozoite surface protein 1 (*pfmsp1*) and *P. falciparum* merozoite surface protein 2 (*pfmsp2*) genes following the WHO-recommended protocol [36]. For both genes, the 20-μl primary reaction consisted of 10 μl of PrimeSTAR Max DNA Polymerase Mix (Takara, Kyoto, Japan), 0.4 μM of each primer, and 3 μl of extracted DNA, and the 10-μl nested reactions consisted of 5 μl of PrimeSTAR Max DNA Polymerase Mix, 0.4 μM of each primer, and 1 μl of product from the primary reaction as template. Nested rounds were conducted separately for each allelic family (*pfmsp1*: MAD20, K1, and RO33; *pfmsp2*: FC27 and 3D7/IC1). PCR primer sequences and cycling conditions are described in Table S1. PCR products from the nested rounds were visualized on 2% agarose gel electrophoresis. Alleles were identified by distinct bands of different sizes, and the multiplicity of infection (MOI) was determined as the higher allele count between *pfmsp1* and *pfmsp2*. Only samples positive for both *pfmsp1* and *pfmsp2* amplifications were included in the *pfhrp2* and *pfhrp3* deletion analysis.

### Evaluation of *pfhrp2 *and *pfhrp3* deletions

Deletions of exons 1 and 2 in *pfhrp2* and *pfhrp3* were determined by nested PCR [36]. Primary and nested PCR reactions followed the same protocol as for *pfmsp1* and *pfmsp2*, with minor adjustments: primer concentration (0.2 μM) and template volume for nested rounds (2 μl of primary product). Primer sequences and cycling conditions are detailed in Table S1.

Nested PCR products were visualized on 2% agarose gel electrophoresis. Since the non-expression of *pfhrp2* and/or *pfhrp3* proteins arises from frameshift mutation caused by exon 1 and exon 2 deletions (region corresponding to the target epitope of *Pf*HRP2-based RDT), samples failing to amplify either or both loci were considered to harbor *P. falciparum* with *pfhrp2* and/or *pfhrp3* deletions. Genomic DNA isolated from cultured *P. falciparum* 3D7 strain (provided by Nagasaki University Institute of Tropical Medicine) served as a positive control for all PCR reactions.

### Data analysis

PCR-confirmed *P. falciparum* infections without fever were considered asymptomatic. *P. falciparum* infections positive by the mitochondrial *cox3* PCR but negative by microscopy were considered submicroscopic. The sensitivity and specificity of RDT and microscopy were determined using PCR as the reference. Mann–Whitney and Kruskal–Wallis tests were used to compare MOI across different groups, while Pearson’s chi-square and Fisher’s exact tests were used to compare proportions among groups. Odds ratios (OR) and associated 95% confidence interval (CI), and probability (*p*) values were generated using logistic regression models to assess the association between the presence of single *pfhrp2* deletion, single *pfhrp3* deletion, and *pfhrp2*/*pfhrp3* double deletions and the following predictor variables: age, sex, MOI, asymptomatic infection, submicroscopic infection, study site, and study period. Multiple imputation was used to handle missing demographic data [37]. Missing values for age and sex were imputed using the linear regression and the logistic regression models, respectively. The imputation procedure included all predictor variables and outcome variables used in the final logistic regression models. Ten imputed datasets were created. The Hosmer–Lemeshow goodness-of-fit test was performed to evaluate the fit of the models for both non-imputed and imputed datasets. *P* < 0.05 were considered statistically significant. Statistical analysis was conducted in Stata 18 (StataCorp, College Station, USA).

## Results

### Study population

A total of 9120 participants were enrolled in this study, with more than 2000 enrolled at each survey time point except January 2019. Of all survey participants, 41.0% were from Mfangano Island, followed by 35.8% from Suba South, 14.8% from Ngodhe Island, and 8.5% from Kibuogi Island. The median age of participants was 9.0 years (IQR 6–12) and 47.9% of participants were male. Approximately 16.0% of all participants had fever, and 49.4% reported LLIN use the night before the survey (Table 1).

**Table 1:** Characteristics of the study population in Homa Bay County, Kenya 2018–2020

	Suba South (*n* = 3261)	Mfangano (*n* = 3742)	Ngodhe (*n* = 1346)	Kibuogi (*n* = 771)	Total (*N* = 9120)
Year–month					
2018–Sep	1352	977	458	208	2995
2019–Jan	200	984	NA	NA	1184
2019–Sep	923	716	442	301	2382
2020–Jan	786	1065	446	262	2559
Sex, *n* (%)^*^					
Male	1591 (48.8)	1726 (46.1)	674 (50.1)	377 (48.9)	4368 (47.9)
Age, Median (IQR)^†^	9.0 (6–11)	9.0 (6–12)	14.0 (6–31)	12.0 (5–27)	9.0 (6–12)
Fever, *n* (%)^‡^					
≥ 37.5 ℃	652 (20.0)	582 (15.6)	147 (10.9)	77 (10.0)	1458 (16.0)
LLIN usage previous night, *n* (%)^§^					
Yes	1493 (45.8)	2007 (53.6)	645 (48.0)	364 (47.2)	4509 (49.4)

*NA* not applicable as Ngodhe and Kibuogi were not surveyed in January 2019 due to an ongoing intervention study

^*^Sex was not recorded for 232 (2.5%) participants

^†^Age was not recorded for 60 (0.7%) participants

^‡^Axillary temperature was missing for 256 (2.8%) participants

^§^LLIN usage was not recorded for 444 (4.9%) participants

### *P. falciparum* infection prevalence and diagnostic performance

Overall *P. falciparum* prevalence by RDT, microscopy, and PCR was 10.9, 6.6, and 15.9%, respectively (Table 2). By all detection methods, prevalence was highest on Mfangano Island and varied significantly (all *p* < 0.001) across sites (Table 2). Of the 1450 *cox3* PCR-positive samples, 852 (58.8%) were microscopy-negative or submicroscopic and 1165 (83.8%) were asymptomatic. A total of 445 (30.7%) RDT-negative and *cox3* PCR-positive samples including 370 submicroscopic infection samples were included in the *P. falciparum* nuclear DNA quality check (Table 2, Fig. 2).

**Table 2:** *P. falciparum* infection prevalence by RDT, microscopy, and PCR, Homa Bay County, Kenya 2018–2020

Diagnostic method	Suba South (*n* = 3261)	Mfangano (*n* = 3742)	Ngodhe (*n* = 1346)	Kibuogi (*n* = 771)	Total (*N* = 9120)	*p* value
RDT, *n* (%)^*^						
Positive	233 (7.1)	690 (18.4)	60 (4.5)	12 (1.6)	995 (10.9)	*p* < 0.001
Negative but PCR positive	116 (3.6)	265 (7.1)	54 (4.0)	10 (1.3)	445 (4.9)	
Microscopy, *n* (%)						
Positive	117 (3.6)	435 (11.6)	38 (2.8)	8 (1.0)	598 (6.6)	*p* < 0.001
PCR, *n* (%)						
Positive	358 (11.0)	956 (25.5)	114 (8.5)	22 (2.9)	1450 (15.9)	*p* < 0.001
Positive but microscopy3 negative	241 (7.4)	521 (13.9)	76 (5.6)	14 (1.8)	852 (9.3)	

*p* values were calculated by the *χ*-square test

^*^RDT results were invalid or missing for 10 participants

Using PCR as reference, the sensitivity and specificity of RDT were 69.1% (95% CI 68.7–69.5) and 94.9% (95% CI 94.5–95.3), respectively, while those of microscopy were 41.2% (95% CI 40.9–41.5) and 91.3% (95% CI 90.9–91.7), respectively.

### *Pfhrp2 *and/or* pfhrp3* deletions

Of the 445 *cox3* PCR-positive but RDT-negative samples, 125 (28.1%) were positive for both *pfmsp1* and *pfmsp2* PCR (Table 3). Most of these 125 infections were submicroscopic (65.6%) and asymptomatic (76.8%). Among these samples, 13 (10.4%), 19 (15.2%), and 36 (28.8%) samples showed single *pfhrp2* deletion single *pfhrp3* deletions, and *pfhrp2/3* double deletions, respectively. Single *pfhrp2* deletion was found in all study sites and cross-sectional surveys, while single *pfhrp3* deletion was found in all sites except Kibuogi, although the number of samples examined was very small (*n* = 3). Of the samples that showed *pfhrp2* and/or *pfhrp3* deletions, most were asymptomatic (80.9%; 55/68) and submicroscopic (73.5%; 50/68) (Table 3).

**Table 3:** *P. falciparum* with *pfhrp2* and *pfhrp3* deletions in Homa Bay County, Kenya 2018–2020

	RDT-negative, *cox3* PCR positive (*N* = 445)	Both *pfmsp1/2* successfully amplified *n*, (%) (*n* = 125)	*Pfhrp2* single deletion (*n* = 13)	*Pfhrp3* single deletion (*n* = 19)	*Pfhrp2/3* double deletion (*n* = 36)	No deletion (*n* = 57)
Survey period						
2018–Sep	132	40 (30.3)	2	7	12	19
2019–Jan	89	22 (24.7)	1	4	5	12
2019–Sep	107	40 (37.4)	5	4	11	20
2020–Jan	117	23 (19.7)	5	4	8	6
Survey place						
Suba South	116	39 (33.6)	1	5	16	17
Mfangano	265	73 (27.5)	10	11	18	34
Ngodhe	54	10 (18.5)	1	3	2	4
Kibuogi	10	3 (30.0)	1	0	0	2
Microscopy						
Negative	370	82 (65.6)	9	14	27	32
Fever						
≥ 37.5 ℃	87	29 (33.3)	2	5	6	16

### *Pfmsp1* and *pfmsp2* allelic families and MOI

For *pfmsp1*, K1-type alleles accounted for 55.5% (81/146) of detected clones, followed by RO33-type and MAD20-type at 27.4% (40/146) and 17.1% (25/146), respectively. For *pfmsp2*, 3D7/IC1-type and FC27-type alleles accounted for 58.4% (80/137) and 41.6% (57/137) of clones, respectively. Between parasites with and without *pfhrp2* and *pfhrp3* deletions, the distributions of allelic families were not significantly different for *pfmsp1* (*p* = 0.94) and *pfmsp2* (*p* = 0.57). Specifically, for *pfmsp1*, among samples with single *pfhrp2* deletions, 7 samples had K1-type alleles, 8 samples had RO33-type alleles, and none had MAD20-type alleles. Among samples with single *pfhrp3* deletions, 13 had K1-type alleles, 4 had MAD20-type alleles, and 5 had RO33-type alleles. In samples with *pfhrp2/3* double deletions, 20 had K1-type alleles, 9 had MAD20-type alleles, and 9 had RO33-type alleles. For *pfmsp2*, 5 samples with single *pfhrp2* deletions had FC27-type alleles, and 9 had IC1-type alleles. Among samples with single *pfhrp3* deletions, 10 had FC27-type alleles, and 12 had IC1-type alleles. In samples with *pfhrp2/3* double deletions, 17 had FC27-type alleles, and 20 had IC1-type alleles (Table 4).

**Table 4:** Distribution of *pfmsp1* and *pfmsp2* allelic families and multiplicity of infection (MOI) in Kenya, 2018–2020

	Single *pfhrp2* deletion	Single *pfhrp3* deletion	*Pfhrp2/3* double deletion	No deletion	*p* value*
*Pfmsp1*					
K1	7	13	20	41	
MAD20	0	4	9	12	
RO33	8	5	9	18	0.84
*Pfmsp2*					
FC27	5	10	17	25	
3D7/IC1	9	12	20	39	0.57
MOI					
1	10	14	31	33	
2	2	4	4	21	
3	0	1	1	3	
4	1	0	0	0	
Mean MOI (SD)	1.38 (0.87)	1.32 (0.58)	1.17 (0.45)	1.47 (0.60)	0.16

^*^Calculated by the Kruskal–Wallis test

Monoclonal (MOI = 1) infections accounted for 70.4% (88/125) of the samples (Table 4). The mean MOI was slightly higher among microscopic infections (1.39 ± SD 0.57) than submicroscopic infections (1.33 ± SD 0.61), although the difference was not statistically significant (*p* = 0.98). The mean MOI was higher in samples with intact *pfhrp2* and *pfhrp3* (1.47 ± SD 0.60) than those with gene deletions (1.38 ± 0.87, 1.32 ± 0.58, and 1.17 ± 0.45 for single *pfhrp2* deletion, single *pfhrp3* deletion, and *pfhrp2/3* double deletions, respectively), although the difference was not significant (*H* = 5.19; *p* = 0.16). Among polyclonal (MOI > 1) infections, *pfhrp2* and/or *pfhrp3* deletions were detected in 35.1% (13/37) of samples, compared to 62.5% (55/88) among monoclonal infections (Table 4).

### Association between *pfhrp2* and *pfhrp3* deletions and predictors

Multiple imputation was used to address missing data for age (7 observations) and sex (1 observation). Neither *pfhrp2* nor *pfhrp3* deletions were statistically significantly associated with any predictor variables. However, polyclonal infections were significantly associated with a lower odds of pfhrp2/3 double deletions (OR = 0.286, *p* = 0.022) (Table 5). The results were similar for imputed and non-imputed models (Table S2).

**Table 5:** Association between predictors and *pfhrp2* and *pfhrp3* deletions in Homa Bay County, Kenya, 2018–2020

	Single *pfhrp2* deletion	Single *pfhrp3* deletion	*Pfhrp2/3* double deletion
Predictors	OR (95% CI)	OR (95% CI)	OR (95% CI)
Age	1.034 (0.981–1.090)	1.008 (0.945–1.074)	0.996 (0.949–1.046)
Sex	0.746 (0.215–2.588)	1.278 (0.427–3.827)	0.880 (0.384–2.016)
Polyclonal infection	0.647 (0.153–2.734)	0.867 (0.251–3.001)	0.286* (0.0981–0.835)
Asymptomatic infection	0.844 (0.151–4.697)	1.678 (0.333–8.455)	1.120 (0.365–3.438)
Submicroscopic infection	1.872 (0.488–7.172)	1.740 (0.501–6.047)	1.761 (0.692–4.483)
Study site	0.557 (0.297–1.045)	1.041 (0.702–1.545)	1.191 (0.876–1.619)
Study period	1.556 (0.853–2.837)	0.932 (0.567–1.532)	1.142 (0.781–1.670)
HL chi2^†^ (*p* value)	5.57 (0.6955)	10.75 (0.2160)	10.69 (0.2199)

^*^p = 0.022

^†^Hosmer–Lemeshow goodness-of-fit test

## Discussion

This study reveals the presence of *pfhrp2/3* double deletions among mostly asymptomatic *P. falciparum* infections in Homa Bay County, western Kenya. Our findings demonstrate that these deletions were found across multiple study sites and survey time points. Most parasites with gene deletions were found in Suba South and Mfangano, where malaria prevalence was relatively high. Infections with *P. falciparum* lacking *pfhrp2* and/or *pfhrp3* were mostly asymptomatic and submicroscopic, highlighting the potential for these parasites to spread undetected.

A previous study [9] reported the presence of *P. falciparum* lacking either *pfhrp2* or *pfhrp3* in Kenya in 2014. We confirm previous findings and report the presence of *P. falciparum* lacking both *pfhrp2* and *pfhrp3* in asymptomatic infections from as early as 2018. Similar observations of *P. falciparum* with *pfhrp2/pfhrp3* double deletions were reported among symptomatic patients in Kilifi County in 2019–2020, a moderate to high malaria transmission setting along the Indian Ocean [23]. Since *Pf*HRP2-based RDTs are widely used as a malaria diagnostic tool in Kenya, the emergence and potential expansion of these parasites with *pfhrp2* and/or *pfhrp3* deletions pose a threat to malaria control and elimination programs in Kenya.

*P. falciparum* with single *pfhrp2* deletion was detected in all four study sites and those with single *pfhrp3* deletion and *pfhrp2/3* double deletions were detected in all sites except Kibuogi. When considered with results from a previous study in the same county reporting *P. falciparum* with either *pfhrp2* or *pfhrp3* deletion [9], our data suggest that the use of *Pf*HRP2-based RDTs might have further selected for parasites most likely to evade detection i.e. *P. falciparum* with *pfhrp2/pfhrp3* double deletions. This is corroborated by our observation that of all samples containing *P. falciparum* with *pfhrp2* and/or *pfhrp3* deletions, more than half harbored *P. falciparum* with *pfhrp2/3* double deletions. Furthermore *P. falciparum* with *pfhrp2/3* double deletions was most frequently observed in Suba South and Mfangano, where parasite prevalence was higher, transmission was more intense [29], and the use of *Pf*HRP2-based RDTs was likely more common. While we previously demonstrated extensive gene flow among *P. falciparum* populations on different islands in the study area [28, 38], the genetic relationships among *P. falciparum* with *pfhrp2* and/or *pfhrp3* deletions identified in this study remain unclear. Other studies have reported that gene deletion strains are more likely to be found within populations with a common genetic background, regardless of malaria prevalence [39].

Of the 125 samples tested for *pfhrp2* and *pfhrp3* deletions in this study, polyclonal infection was more common in samples with intact *pfhrp2* and *pfhrp3*. Our findings align with studies from Cameroon, India [40], and South Sudan [41]. It is possible that in polyclonal infections, parasites with gene deletions were co-infected with those with intact *pfhrp2* and/or *pfhrp3*. Successful PCR amplification of *pfhrp2* and/or *pfhrp3* from the latter parasites might have masked the presence of the former. Furthermore, co-infection of *P. falciparum* with deleted and intact *pfhrp2 and pfhrp3* could have led to positive RDT diagnosis, which was excluded in our analysis. Our PCR-based detection methodology and sample selection strategy likely underestimated the number of samples containing *P. falciparum* with *pfhrp2/pfhrp3* double deletions.

Our previous study [26] and this study indicate that most *P. falciparum* infections in the study area were submicroscopic and asymptomatic, which represented a hidden reservoir to sustain transmission, because individuals with these infections were unlikely to seek diagnosis and treatment in health facilities. Since *P. falciparum* in these asymptomatic infections were not under selection by *Pf*HRP2-based RDTs, detection of parasites with *pfhrp2* and/or *pfhrp3* deletions, especially those with double deletions in our samples was unexpected. Nair et al. [42] used competitive growth assays to demonstrate the substantial fitness cost incurred by single *pfhrp2* deletion and *pfhrp2/3* double deletions, which implies that in our study area with co-circulating *P. falciparum* with intact and deleted *pfhrp2* and/or *pfhrp3*, those with deletions would likely die out especially among asymptomatic (and undiagnosed) infections. However, we observed *P. falciparum* with *pfhrp2/3* double deletions in all study sites except Kibuogi throughout the study period. In addition, a mathematical model has identified Kenya as a country at high risk for the emergence and spread of *P. falciparum* with *pfhrp2/3* double deletions [43]. Therefore, systematic evaluation of the diagnostic performance of *Pf*HRP2-based RDTs is warranted.

A recent report from Ethiopia [44] indicated that a *kelch13* (*K13*) mutation that confers partial resistance to artemisinin was found more frequently in *P. falciparum* with *pfhrp2* and/or *pfhrp3* deletions, suggesting a potentially different mechanism by which *pfhrp2* and/or *pfhrp3* deletions might be maintained in a population of mixed parasites with intact and deleted *pfhrp2* and/or *pfhrp3*, as in our study area. No evidence of artemisinin resistance in *P. falciparum* from the study area was found in our previous study [45], however artemisinin-resistant *P. falciparum* have emerged in neighboring Uganda [46, 47] and Tanzania [48], and in the wider Great Lake region of east Africa [49, 50]. Cross-border movement likely resulted in the introduction of artemisinin-resistant *P. falciparum* from Uganda to Busia County, Kenya [51]. While challenges and initiatives to control “border malaria” have been well documented, coordinated policies among east African nations may be urgently required to respond to the potential emergence and spread of drug- and diagnostic-resistant *P. falciparum* [52].

This study has several limitations. Most of the PCR-confirmed *P. falciparum* infections in this study were submicroscopic, therefore only about nine percent of all infections were included in the analysis of *pfhrp2* and *pfhrp3* deletions, and the prevalence of parasites with deletions could not be determined. This study used the conventional PCR-based protocol to detect *pfhrp2* and *pfhrp3* deletions [36], which was originally developed for symptomatic malaria cases with higher parasitemia. More sensitive analytical methods based on qPCR [53, 54] and droplet digital PCR (ddPCR) [55] have recently been developed that may overcome the challenges of detecting *pfhrp2* and/or *pfhrp3* deletions in low-density and polyclonal infections. No breakpoint analysis was performed to determine the extent to which *pfhrp2* and *pfhrp3* were deleted, and no genetic/genomic analysis was conducted to examine if *P. falciparum* with *pfhrp2* and/or *pfhrp3* deletions from different study sites and different years shared similar genetic backgrounds.

## Conclusions

We demonstrated the presence of *P. falciparum* with single *pfhrp2* deletion*,* single *pfhrp3* deletion*,* and *pfhrp2/3* double deletions among asymptomatic infections in western Kenya. These findings indicate the need to enhance active molecular surveillance of *pfhrp2 and pfhrp3* deletions to monitor the performance of *Pf*HRP2-based RDTs to ensure effective malaria control and elimination. Further research is needed to understand the genetic relationships among parasites with *pfhrp2* and *pfhrp3* deletions, their prevalence and impact on malaria transmission dynamics.

## Supplementary Information

Additional file 1.

## Data Availability

The datasets used and analyzed during the current study are available from the corresponding author on reasonable request.

## Abbreviations

RDT: Rapid diagnosis test
WHO: World Health Organization
*Pf*HRP2: *Plasmodium falciparum* Histidine Rich Protein 2
*Pf*HRP3: *Plasmodium falciparum* Histidine Rich Protein 3
LLIN: Long-lasting insecticidal net
IRS: Indoor residual spray
DBS: Dried blood spot
PCR: Polymerase chain reaction
*cox3*: Cytochrome c oxidase III
*pfmsp1*: *Plasmodium falciparum* Merozoite surface protein 1
*pfmsp2*: *Plasmodium falciparum* Merozoite surface protein 2
MOI: Multiplicity of infection
