# Supplementary material for: Plasmodium falciparum with pfhrp2 and pfhrp3 gene deletions in asymptomatic malaria infections in the Lake Victoria region, Kenya
Source: Trop Med Health. 2024 Dec 18;52:94. doi: 10.1186/s41182-024-00664-7 (PMC11653864; doi:10.1186/s41182-024-00664-7)
Supplement: Supplementary file 1 — Additional file 1. [file 41182_2024_664_MOESM1_ESM.docx]

**Supplemental Documents**

**Table S1. PCR primers and cycling conditions for *pfmsp1/2* and *pfhrp2/3***

| Target | F/R | 1st/2nd | Primer sequence (5'-3') | Amplification length from nested (bp) | Annealing temperature | Cycle condition |
| --- | --- | --- | --- | --- | --- | --- |
| *merozoite surface protein 1* | M1-OF | 1st | CTAGAAGCTTTAGAAGATGCAGTATTG |  | 65 | 98 ℃ 10 sec - 68 ℃ 30 sec [35 cycles] 4 ℃ |
|  | M1-OR | 1st | CTTAAATAGTATTCTAATTCAAGTGGATCA |  |  |  |
|  | M1-KF | 2nd | AAATGAAGAAGAAATTACTACAAAAGGTGC | 241 | 77 | 98 ℃ 10 sec - 70 ℃ 30 sec [30 cycles] 4 ℃ |
|  | M1-KR | 2nd | GCTTGCATCAGCTGGAGGGCTTGCACCAGA |  |  |  |
|  | M1-MF | 2nd | AAATGAAGGAACAAGTGGAACAGCTGTTAC | 187 | 73 | 98 ℃ 10 sec - 70 ℃ 30 sec [30 cycles] 4 ℃ |
|  | M1-MR | 2nd | ATCTGAAGGATTTGTACGTCTTGAATTACC |  |  |  |
|  | M1-RF | 2nd | TAAAGGATGGAGCAAATACTCAAGTTGTTG | 153 | 76 | 98 ℃ 10 sec - 70 ℃ 30 sec [30 cycles] 4 ℃ |
|  | M1-RR | 2nd | CATCTGAAGGATTTGCAGCACCTGGAGATC |  |  |  |
| *merozoite surface protein 2* | M2-OF | 1st | ATGAAGGTAATTAAAACATTGTCTATTATA |  | 59 | 98 ℃ 10 sec - 57 ℃ 5 sec - 70 ℃ 30 sec [30 cycles] 4 ℃ |
|  | M2-OR | 1st | CTTTGTTACCATCGGTACATTCTT |  |  |  |
|  | M2-FCF | 2nd | AATACTAAGAGTGTAGGTGCARATGCTCCA | 434 | 72 | 98 ℃ 10 sec - 70 ℃ 30 sec [23 cycles] 4 ℃ |
|  | M2-FCR | 2nd | TTTTATTTGGTGCATTGCCAGAACTTGAAC |  |  |  |
|  | M2-ICF | 2nd | AGAAGTATGGCAGAAAGTAAKCCTYCTACT | 473 | 72 | 98 ℃ 10 sec - 70 ℃ 30 sec [23 cycles] 4 ℃ |
|  | M2-ICR | 2nd | GATTGTAATTCGGGGGATTCAGTTTGTTCG |  |  |  |
| *histidine rich protein 2 exon2* | F | 1st | CAAAAGGACTTAATTTAAATAAGAG | 791 | 1st: 48 | 98 ℃ 10 sec - 48 ℃ 5 sec - 70 ℃ 20 sec [40 cycles] 4 ℃ |
|  | R | 1st, 2nd | AATAAATTTAATGGCGTAGGCA |  | 2nd: 71 | 98 ℃ 10 sec - 71 ℃ 45 sec [38 cycles] 4 ℃ |
|  | F | 2nd | ATTATTACACGAAACTCAAGCAC |  |  |  |
| *histidine rich protein 2 exon1-exon2* | F | 1st | GGT TTC CTT CTC AAA AAA TAA AG | 223 | 1st: 58 | 95 ℃ 10 sec - 58 ℃ 15 sec - 72 ℃ 5 sec [30 cycles] 4 ℃ |
|  | R | 1st | TCT ACA TGT GCT TGA GTT TCG |  | 2nd: 66 | 95 ℃ 10 sec - 66 ℃ 15 sec - 72 ℃ 5 sec [30 cycles] 4 ℃ |
|  | F | 2nd | GTA TTA TCC GCT GCC GTT TTT GCC |  |  |  |
|  | R | 2nd | CTA CAC AAG TTA TTA TTA AAT GCG GAA |  |  |  |
| *histidine rich protein 3 exon2* | F | 1st | AATGCAAAAGGACTTAATTC | 699 | 1st: 49 | 98 ℃ 10 sec - 49 ℃ 5 sec - 70 ℃ 20 sec [40 cycles] 4 ℃ |
|  | R | 1st, 2nd | TGGTGTAAGTGATGCGTAGT |  | 2nd: 51 | 98 ℃ 10 sec - 51 ℃ 5 sec - 72 ℃ 20 sec [18 cycles] 4 ℃ |
|  | F | 2nd | AAATAAGAGATTATTACACGAAAG |  |  |  |
| *histidine rich protein 3 exon1-exon2* | F | 1st | GGT TTC CTT CTC AAA AAA TAA AA | 226 | 1st: 57 | 95 ℃ 10 sec - 57 ℃ 5 sec - 72 ℃ 5 sec [30 cycles] 4 ℃ |
|  | R | 1st | ATA TTA TCC GCT GCC GTT TTT GCT |  | 2nd: 60 | 95 ℃ 10 sec - 60 ℃ 5 sec - 72 ℃ 5 sec [30 cycles] 4 ℃ |
|  | F | 2nd | CCT GCA TGT GCT TGA CTT TC |  |  |  |
|  | R | 2nd | CTA AAC AAG TTA TTG TTA AAT TCG GAG |  |  |  |

**Logistic Regression**

The statistical analysis aimed to investigate any association between *pfhrp2*, *pfhrp3*, and double deletions with **multiplicity of infection (MOI) type, age, sex, asymptomatic infection, submicroscopic infection, study site, and survey period** was conducted in Stata 18.5 (StataNow, StataCorp. 2024. College Station, TX: StataCorp LLC)

## **Variable Definitions**

**Outcomes**

double_deletion: Binary outcome variable indicating presence of *pfhrp2*/3 double deletions (1 = double deletion, 0 = no double deletion).

*pfhrp2*_num: Binary outcome variable indicating presence of *pfhrp2* deletion (1 = *pfhrp2* deletion, 0 = no *pfhrp2* deletion).

*pfhrp3*_num: Binary outcome variable indicating presence of *pfhrp3* deletion (1 = *pfhrp3* deletion, 0 = no *pfhrp3* deletion).

**Predictors**

**Age**: Continuous variable representing the age of the survey participants.

**Sex**: Binary variable indicating sex of the survey participants (1 = male, 0 = female).

**Polyclonal infection**: Binary variable indicating type of multiplicity of infection (1 = polyclonal, 0 = monoclonal).

**Asymptomatic infection**: Binary variable indicating asymptomatic infection (1 = asymptomatic, 0 = Symptomatic) was based on febrile status, defined as axillary temperature of >= 37.5 degree Celsiusmic

**Submicroscopic infection:** Binary variable indicating submicroscopic infection (1 = submicroscopic, 0 = microscopic) was based malaria results of malaria light microscopy

**Study site**: Categorical variable indicating study site, converted to numerical codes for the four study sites

**Study period**: Categorical variable indicating survey period, converted to numerical codes for the four surveys

## **Logistic Regression Models**

Logistic regression models were employed to evaluate the associations between the presence of deletions (*pfhrp2*, *pfhrp3*, or both) and various predictor variables, including age, sex, multiplicity of infection (MOI), asymptomatic infection, submicroscopic infection, study site, and study period. The log odds of observing deletions were modeled as a linear combination of these predictors, with coefficients estimated using maximum likelihood estimation. This analysis was performed using the logistic command in STATA version 18.5, presenting the model estimates as odds ratios (ORs). For each predictor, 95% confidence intervals (CIs) were calculated based on the standard errors of the estimated coefficients to assess the precision of the ORs. P-values were generated via Wald tests. A p-value of <0.05 was considered statistically significant. Model fit was evaluated using the Hosmer–Lemeshow goodness-of-fit test, implemented in STATA with the estat gof, group(10) command.

## **Model parameterization:**

**Double Deletion(DD):**

$$\text{Logit}(P(\text{DD}=1))=\beta_{0}+\beta_{1}\text{age}+\beta_{2}\text{sex\_num}+\beta_{3}\text{moi\_type\_num}+\beta_{4}\text{asymptomatic infection}+\beta_{5}\text{submicroscopic infection}+\beta_{6}\text{ssite\_num}+\beta_{7}\text{studyperiod}$$

***Pfhrp2* deletion:**

$$\text{Logit}(P(pf\text{hrp2}=1))=\beta_{0}+\beta_{1}\text{age}+\beta_{2}\text{sex\_num}+\beta_{3}\text{moi\_type\_num}+\beta_{4}\text{asymptomatic infection}+\beta_{5}\text{submicroscopic infection}+\beta_{6}\text{ssite\_num}+\beta_{7}\text{studyperiod}$$

***Pfhrp3* deletion:**

$$\text{Logit}(P(pf\text{hrp3}=1))=\beta_{0}+\beta_{1}\text{age}+\beta_{2}\text{sex\_num}+\beta_{3}\text{moi\_type\_num}+\beta_{4}\text{asymptomatic infection}+\beta_{5}\text{submicroscopic infection}+\beta_{6}\text{ssite\_num}+\beta_{7}\text{studyperiod}$$

Where:

•P(DD=1) or P(*pfhrp2*=1) or P(*pfhrp3*=1) is the probability of the deletion

•β_0_ is the intercept

•β_1_-β_7_ represent are the coefficients for each independent variable quantifying their association with the log odds of the outcome. A positive coefficient means that an increase in the predictor variable is associated with an increase in the log odds of the outcome (and thus an increased probability). A negative coefficient means the opposite.

## **Model outputs**

In analysis, multiple imputation was utilized to address missing data for age and sex, to ensure robust and unbiased logistic regression analyses. There was 1 missing observation for age and 7 missing observations for sex. These missing data were due to non-responses at the request of the participants and unintentional omissions at the time of data collection. The imputed models generally showed similar odds ratios and confidence intervals compared to models without imputation, indicating that the imputation process effectively handled missing data without significantly altering the results (Table S2). Key findings include the identification of polyclonal infection as a significant predictor for double deletion in both imputed (OR: 0.293, 95% CI: 0.101 - 0.851) and non-imputed models (OR: 0.286, 95% CI: 0.0981 - 0.835).

## **Table S2 Logistic regression models with and without multiple data imputation**

|  | Double deletion | | *pfhrp2* | | *Pfhrp3* | |
| --- | --- | --- | --- | --- | --- | --- |
|  | Without imputation n=117 | With imputation n=125 | Without imputation n=117 | With imputation n=125 | Without imputation n=117 | With imputation n=125 |
| Predictors | *OR*  *95% CI* | *OR*  *95% CI* | *OR*  *95% CI* | *OR*  *95% CI* | *OR*  *95% CI* | *OR*  *95% CI* |
| **Age** | 0.996 | 0.995 | 1.034 | 1.032 | 1.008 | 1.007 |
|  | (0.949 - 1.046) | (0.947 - 1.045) | (0.981 - 1.090) | (0.979 - 1.087) | (0.945 - 1.074) | (0.945 - 1.073) |
| **Sex** | 0.880 | 1.027 | 0.746 | 0.849 | 1.278 | 0.979 |
|  | (0.384 - 2.016) | (0.452 - 2.331) | (0.215 - 2.588) | (0.240 - 3.003) | (0.427 - 3.827) | (0.361 - 2.655) |
| **Polyclonal infection** | 0.286* | 0.293** | 0.647 | 0.641 | 0.867 | 0.868 |
|  | (0.0981 - 0.835) | (0.101 - 0.851) | (0.153 - 2.734) | (0.152 - 2.702) | (0.251 - 3.001) | (0.280 - 2.687) |
| **Asymptomatic** | 1.120 | 1.675 | 0.844 | 1.447 | 1.678 | 0.838 |
|  | (0.365 - 3.438) | (0.575 - 4.877) | (0.151 - 4.697) | (0.267 - 7.837) | (0.333 - 8.455) | (0.259 - 2.711) |
| **Submicroscopic** | 1.761 | 1.604 | 1.872 | 1.691 | 1.740 | 1.587 |
|  | (0.692 - 4.483) | (0.634 - 4.060) | (0.488 - 7.172) | (0.446 - 6.403) | (0.501 - 6.047) | (0.511 - 4.928) |
| **Study site** | 1.191 | 1.269 | 0.557 | 0.593 | 1.041 | 0.946 |
|  | (0.876 - 1.619) | (0.939 - 1.715) | (0.297 - 1.045) | (0.319 - 1.102) | (0.702 - 1.545) | (0.653 - 1.371) |
| **Study period** | 1.142 | 1.173 | 1.556 | 1.638 | 0.932 | 0.895 |
|  | (0.781 - 1.670) | (0.798 - 1.724) | (0.853 - 2.837) | (0.883 - 3.038) | (0.567 - 1.532) | (0.557 - 1.436) |
| Constant | 0.712 | 0.375 | 0.112 | 0.0534 | 0.0713 | 0.244 |
|  | (0.0784 - 6.467) | (0.0456 - 3.087) | (0.00469 - 2.699) | (0.00254 - 1.124) | (0.00357 - 1.424) | (0.0212 - 2.805) |

*P value = 0.22; ** 0.24
